# Isolation and Pathogenicity of a Natural Recombinant Pig Reproductive and Respiratory Syndrome Virus in Northeast China

**DOI:** 10.3390/v17050729

**Published:** 2025-05-19

**Authors:** Zhixin Tian, Qiwei Li, Luxiang Xu, Dexin Liang, Yuan Li, Ziqi Shi, Lingzhi Luo, Jiechao Jin, Xiaoyi Huo, Xiumei Dong, Han Zhou

**Affiliations:** 1 College of Veterinary Medicine, Northeast Agricultural University, Harbin 150030, China; s220602032@neau.edu.cn (Z.T.); qiweili2001@163.com (Q.L.); a15153366354@163.com (L.X.); liangdxdn@163.com (D.L.); szq13115449695@163.com (Z.S.); llz19952021@163.com (L.L.); 15093056525@163.com (J.J.); 17643226950@163.com (X.H.); 2 Heilongjiang Key Laboratory for Animal Disease Control and Pharmaceutical Development, Harbin 150030, China

**Keywords:** NADC 34-like PRRSV, NADC30-like PRRSV, recombination, pathogenicity

## Abstract

First reported in 1987, the porcine reproductive and respiratory syndrome virus (PRRSV) has significantly disrupted the major regions affected by PRRSV in the pig breeding industry. Recently, outbreaks of disease caused by recombinant PRRSV strains in China have raised serious concerns. Effective immunization and infection control in pig populations is critical, as the virus frequently undergoes mutation and recombination. This study characterized a novel recombinant PRRSV strain, BX/CH/22, isolated from Northeast China. Genetic analysis revealed that BX/CH/22 is a recombinant of JXA1, NADC 30-like, and NADC 34-like strains. Phylogenetic analysis of the non-structural protein (NSP) 2 region classified BX/CH/22 as JXA1 PRRSV-like, with a characteristic deletion of 30 discontinuous amino acids in NSP2. However, Open Reading Frame (ORF) 5 analysis classified it as NADC 30-like PPRSV, while whole-genome phylogenetic analysis classified it as NADC 34-like PPRSV. Recombination analysis revealed that BX/CH/22 contains an NADC 34-like PRRSV backbone, an NSP-coding region from NADC 30-like PRRSV, and an ORF2-ORF6 region from NADC 34-like PRRSV. The strain was isolated from serum samples obtained from commercial swine farms undergoing active PRRS outbreaks. In animal experiments, all BX/CH/22-challenged piglets exhibited persistent fever, with peak temperatures >40.5 °C at 4–9 dpi resolving by 11 dpi, accompanied by cough, anorexia, and lethargy. A significant reduction in daily weight gain was observed in infected groups compared to asymptomatic controls, with a 100% survival rate. Our findings provide early warning for PRRSV immune control strategies.

## 1. Introduction

Porcine reproductive and respiratory syndrome (PRRS) was first discovered in North America in 1987 [1]; it has now become a major disease affecting the major regions affected by PRRSV in the pig breeding industry [2]. The PRRS virus (PRRSV; genus, *Porartevirus*; family, Arteriviridae; order, Nidovirales) is a 15.4 kb single-stranded RNA virus with an outer envelope [3,4]. The PRRSV genome includes 10 open reading frames (ORFs): ORF 1 a, ORF 1 b, ORF 2 a, ORF 2 b, ORF 3, ORF 4, ORF 5, ORF 5 a, ORF 6, and ORF 7 [5]. PRRSV is phylogenetically classified into the following two distinct genotypes: PRRSV-1 (European prototype, Betaarterivirus suid 1) and PRRSV-2 (North American prototype, Betaarterivirus suid 2), exhibiting approximately 40–60% whole-genome nucleotide sequence divergence [6,7]. Based on the analysis of 82,237 ORF5 sequences reported worldwide from 1989 to 2021, PRRSV-2 was divided into 11 inherited lineages (L1-L11) and 21 sublineages (L1A-L1F, L1H-L1J, L5A-L5B, L8A-L8E, and L9AL9E) [8,9]. Since its emergence in the late 1990s, PRRSV-2 has become prevalent in China and has evolved rapidly [10]. The emerging epidemiological ascendancy of PRRSV-2 is mechanistically linked to accelerated evolutionary dynamics characterized by frequent recombination events and mutation-driven antigenic diversification, thereby enhancing viral adaptation and population persistence in swine hosts [11]. PRRSV-2 lineage 1.5 has exhibited rapid nationwide expansion through quasi-species evolution and recombination, generating virulent antigenic variants that intensified PRRS outbreaks with severe reproductive-respiratory pathology [12]. In China, novel recombinant NADC30-like PRRSV strains exhibit a different pathogenesis from previous strains [13]. In this study, one recombinant PRRSV strain, BX/CH/22, was isolated from a PRRSV infection in Northeast China. We performed whole-genome sequencing of this PRRSV-2 lineage 1.5 isolate and systematically evaluated its pathogenicity in piglets through clinical monitoring, virological (viral load quantification) and immunological (neutralizing antibody kinetics) profiling, and histopathological assessment. The observed virulence phenotypes and molecular characteristics suggest genomic variations in this strain may correlate with pathobiological adaptations, warranting further comparative pathogenesis studies with contemporary field strains.

## 2. Materials and Methods

### 2.1. Sample Collection and Genome Sequencing

Between 2022 and 2024, we collected 2580 serum samples with suspected infections and 900 lung tissue samples from eight pig farms in Northeast China. During this period, severe reproductive disorders in pregnant sows and respiratory issues in piglets during suckling and after weaning occurred frequently. All eight farms maintained structured immunization protocols using CH-1R antigen-strain vaccines, yet exhibited clinical profiles suggestive of field virus incursion during restocking phases. All samples were kept in iceboxes and promptly transported to the laboratory. Upon arrival, they were stored at −80 °C until analysis. Total RNA was extracted from the positive serum samples using the Qiagen RNeasy Mini kit (Qiagen, Hilden, Germany). cDNA was synthesized with Oligo dT primers according to the manufacturer’s instructions (TaKaRa, Dalian, China). For Nsp2 amplification, the forward primer was 5′-AAGGTCAGATCCGATTG-3′, and the reverse primer was 5′-CTGTGAGGACG-CAGACA-3′. Polymerase chain reaction (PCR) cycling conditions were 95 °C for 4 min, followed by 30 cycles of 94 °C for 1 min, 58 °C for 30 s, and 72 °C for 30 s, with a final 72 °C extension for 10 min, yielding a 563 bp Nsp2 gene fragment. For GP5 gene amplification, the primers were 5′-CATGACACCTGAGACYATGMGGTGG-3′ and 5′-CAWGAGTAGCGCCAGGACATGC-3′. The PCR cycling conditions were 95 °C for 30 s, followed by 40 cycles of 95 °C for 5 s and 60 °C for 20 s. The amplification yielded the complete sequence of the GP5 gene. The PCR products were then purified using a PCR purification kit (Axygen, Silicon Valley, CA, USA) and cloned into the pMD 18-T vector (TaKaRa, China). At least three clones from each cDNA fragment were generated and used for sequencing. Sanger sequencing was performed by Biological Biotechnology (Shanghai, China), and the obtained sequences were assembled using Lasergene software DNAstar 7.1 (DNASTAR, Inc., Shanghai, China).

### 2.2. Sequence Analysis and Phylogenetic Analysis

Amino acid sequence alignment was performed using the ClustalW algorithm in MegAlign (Lasergene, DNASTAR, Inc.). The ORF5 and whole-genome sequences of the reference strains from the GenBank database, along with the PRRSV strain obtained in this study, were aligned using MEGA 7.0. Molecular evolution analysis was conducted in MEGA 7.0, and a phylogenetic tree was constructed using the neighbor-joining method with 1000 bootstrapping replicates. Recombination analysis was performed using the RDP4 software (version RDP4.46); here, potential recombination events were assessed through seven algorithms (RDP, GeneConv, BootScan, MaxChi, Chimera, SiScan, and 3 Seq) with Bonferroni corrections. A recombination event was considered valid if detected by at least four methods in RDP 4 [14]. Representative L1A (IA/2014/NADC34, Accession No. MF326985.1), L1C (NADC30, Accession No. JN654459.1), L8.7 (JXA1, Accession No. EF112445.1), L3.5 (QYYZ, Accession No. JQ308798.1), and L5.1 (VR2332, Accession No. EF536003.1) were used as reference strains to analyze recombination patterns. Strains not involved in recombination were excluded to determine the final recombination location. Recombination events were identified using Simplot v. 3.5.1.

### 2.3. Isolation and Identification

Serum (*n* = 2580) and lung tissue (*n* = 900) specimens were systematically collected from eight CH-1R-vaccinated farms during active outbreaks exhibiting acute respiratory signs. Antigen-antibody detection was performed using ELISA and RT-PCR, resulting in 136 positive samples. Post-quality control, virological isolation from epidemiologically linked specimens confirmed seven isolates that were classified as genotype 2 PRRSV, each derived from distinct biological sources. Genetic evolution analysis of some genes from these strains led to the selection of one with a unique recombination pattern. The positive serum was filtered through a 0.22 µm membrane, and 1 mL aliquots were inoculated into porcine alveolar macrophage (PAM) cells under sterile conditions. The cultures were incubated at 37 °C with 5% CO_2_ for 1 h. Primary porcine alveolar macrophages (PAMs), isolated via bronchoalveolar lavage, were maintained in DMEM (Thermo Fisher, Waltham, MA, USA) supplemented with 10% FBS and passaged three times by standard trypsinization onto fresh culture plates [15]. The infected cells underwent two freeze-thaw cycles at −80 °C, and the supernatant was centrifuged at 5000× *g* for 5 min before being transferred to MARC-145 cells (Thermo Fisher, Shanghai, China) for five consecutive passages and monitored for cytopathic effect (CPE). The supernatants of the positive viral isolates were tested for PRRSV using indirect immunofluorescence (IFA) with a PRRSV N protein-specific monoclonal antibody (GeneTex, Irvine, CA, USA) and fluorescein isothiocyanate (FITC)-conjugated secondary antibodies (Proteintech, Wuhan, China). The viral progeny from three sequential blind passages in porcine alveolar macrophages (PAMs) were harvested and subsequently propagated in MARC-145 cells. Infected MARC-145 cells were incubated for 48 h, then fixed in 4% paraformaldehyde for 10 min and permeabilized with 0.2% Triton X-100 for 5 min. After blocking with 5% bovine serum albumin (BSA) at 37 °C, the cells were incubated overnight at 4 °C with a PRRSV N protein-specific monoclonal antibody (1:500 dilution), followed by incubation with a FITC-conjugated secondary antibody (1:10,000 dilution) for 1 h at 37 °C. The nuclei were counterstained with 4′,6-diamino-2-phenylindole (DAPI). The cells were then viewed with a fluorescence microscope, and those with green fluorescence in the cytoplasm were considered positive. The viral isolate was subsequently subjected to whole-genome sequencing using an established next-generation Illumina platform. The TCID50 of PRRSV was determined via the Reed-Muench corrected endpoint method using ten-fold dilution series of viral inoculum replicated in MARC-145 cell-seeded 96-well plates, with CPE as the scoring criterion [16].

Growth kinetics analysis of isolates: PRRSV inoculum (5 × 10^5.5^ genomic copies/mL) was adsorbed onto 80% confluent MARC-145 monolayers using established adsorption methodology. Cell culture supernatants were collected at 0, 24, 48, 72, 96, 120, and 144 h post-infection. Viral titers were determined using the TCID_50_ method. Cell supernatants were subjected to 10-fold serial dilutions (10^−1^ to 10^−8^) and were seeded into eight replicate wells per dilution. After incubation at 37 °C for 72 h, viral replication was detected by IFA using an anti-PRRSV N protein monoclonal antibody (Median Diagnostics, Beijing, China, 1:200 dilution) as the primary antibody and a FITC-conjugated goat anti-mouse IgG (Sigma, Shanghai, China, 1:500 dilution) as the secondary antibody. The Reed-Muench method was used to calculate the TCID_50_/mL. Transmission electron microscopy (TEM) photography was performed on the viral samples.

### 2.4. Animal Experiments

The PRRSV-antigen/antibody-free piglets from conventional farms used in this study were maintained in strict accordance with the general requirements for biosafety of laboratory animal facilities (GB/T 27416-2014 [17]) and the guidelines for the care and use of laboratory animals (NIH Publication No. 85-23). The animals were housed in a controlled environment with a physical isolation system. The challenge and control groups were kept in separate animal rooms, each equipped with an independent ventilation cage (IVC) system (Tecniplast, Buguggiate, Italy). The air handling system included a three-stage filtration device, and a one-way flow design was implemented to maintain biosafety. Eight 4-week-old piglets, confirmed negative for PRRSV, pseudorabies virus (PRV), and classical swine fever virus (CSFV), were randomly allocated into challenge and control groups (*n* = 4 each) and raised in group-housed cohorts under biosafety containment. The challenge group received 2 mL intramuscular and 2 mL intranasal inoculations of the BX/CH/22 (1 × 10^5 TCID50^/mL), while the control group received DMEM under the same conditions. Rectal temperature and clinical signs in animals were recorded daily, and body weight was measured every other day. Blood samples were collected at 0, 1, 3, 5, 7, 9, 11, and 14 days post-infection (dpi), and the viremia was quantified by in-house probe-based RT-qPCR (standard curve: Y = −2.948X + 36.07, R^2^ = 0.9993). At 14 dpi, all piglets were euthanized, and tissue samples from the heart, lungs, submandibular lymph nodes, tonsils, and brain were collected for RT-qPCR analysis. The severity of lung injury in PRRSV-infected piglets was evaluated using a standardized quantitative evaluation system based on the “Pathological Scoring Guidelines for Animal Disease Models” [18]. Double-blind histological readings were performed, and the mean lesion area was determined after three independent verifications. The lobe weight allocation system was established according to the anatomical volume proportions of the lung lobes, with the following weight coefficients: the right apical lobe (RC), right heart lobe, left apical lobe, and left diaphragmatic lobe each accounted for 10% of the weight coefficient; the accessory lobe accounted for 5%; and the total functional retention of the left and right lungs accounted for 27.5% each. The total scoring domain value was set to 100 points, corresponding to the functional integrity of the entire lung tissue. Histopathological injury grading criteria were as follows: Lung tissue sections were stained with hematoxylin and eosin (H&E) and evaluated according to the recommended standards of the International Veterinary Pathological Society (IVPS) [19]. Grade 0 indicated an intact lung tissue structure with no characteristic pathological changes. Grade 1 was defined by localized interstitial thickening with monocytic infiltration affecting ≤20% of alveolar areas. Grade 2 involved multifocal inflammatory cell infiltration with type I alveolar epithelial cell detachment, affecting 21–40% of the lung parenchyma. Grade 3 was characterized by confluent interstitial pneumonia with type II alveolar epithelial cell hyperplasia, involving 41–60% of the lung tissue. Lastly, Grade 4 represented severe pathology, with diffuse alveolar septal fibrosis and hyaline membrane formation affecting >60% of the lung parenchyma.

For the histopathological analysis, lung samples were collected from each piglet at 14 dpi, fixed in 4% formaldehyde, and stained using H&E. Additionally, the lung sections were subjected to immunohistochemical staining. The tissue sections were first treated with a 3% hydrogen peroxide solution (pH 7.6) for 10 min, followed by incubation with 5% BSA (Thermo Fisher, Shanghai, China) for 30 min. The sections were treated with a rabbit monoclonal antibody against the PRRSV nucleocapsid protein (1:500) as the primary antibody. After three washes with phosphate-buffered saline, the sections were incubated with biotin-conjugated affinity-purified goat anti-rabbit immunoglobulin G as the secondary antibody for 30 min at 37 °C. After further incubation with streptavidin and biotin complex for 30 min at 37 °C, specific antibody binding to the sections was visualized with diaminobenzidine.

### 2.5. Serological Testing

PRRSV-specific antibodies were assessed using a commercially available ELISA kit (IDEXX, Inc., Westbrook, ME, USA) according to the manufacturer’s instructions. According to the manufacturer’s guidelines, sample-to-positive control (S/P) ratios exceeding 0.4 were considered indicative of a positive result.

### 2.6. Statistical Analysis

Hypothesis-driven pairwise comparisons (GraphPad Prism 6.0) incorporated α-error control for longitudinal datasets, with significance threshold adjusted to *p* < 0.05 (two-tailed).

## 3. Results

### 3.1. Virus Isolation and Identification

**Figure 1:**
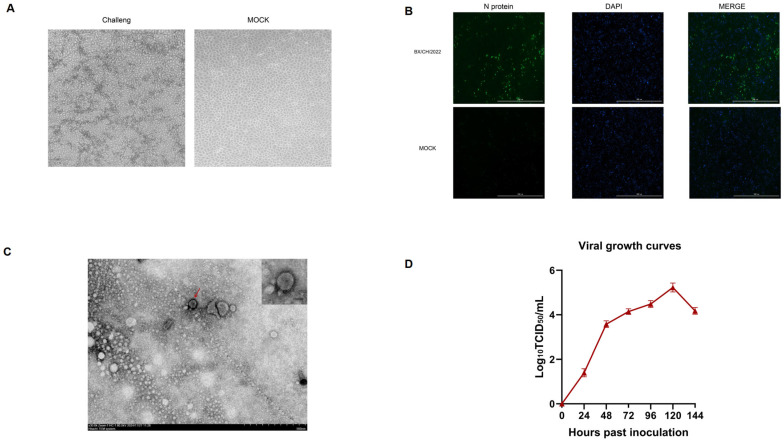
Isolation and identification of the BX/CH/22 strain. (**A**) Cytopathic effects in PRRSV-adapted MARC-145 cells at indicated timepoints. (**B**) N protein-specific immunofluorescence in PRRSV-infected MARC-145 cells versus mock controls (antibody details are presented in the Material and Methods section). (**C**) Electron micrograph showing the viral particles in isolates. The red arrow indicates the location of the virion. (**D**) Viral growth curves.

PRRSV isolation was achieved through sequential propagation in porcine alveolar macrophages (PAM, passage 3) followed by adaptation in MARC-145 cells (passage 5) under serum-free conditions. Typical cytopathic effects (CPEs) first emerged post-adaptation, progressing temporally from sparse foci at 24 hpi to confluent patterns at 72 hpi (Figure 1A). Following triple PAM passages and MARC-145 adaptation (5×), BX/CH/22-derived virus demonstrated sustained CPE with rising RNA titers, validated via nucleocapsid-specific IFA confirming productive replication. (Figure 1B); TEM of PRRSV isolates revealed spherical particles (50–70 nm) with distinct surface glycoprotein projections, confirming ultrastructural integrity consistent with Arteriviridae morphology (Figure 1C) versus mock-infected controls. Electron microscopy revealed characteristic arteriviral particles (≈50–65 nm; Figure 1C). Multistep growth analysis (peak 1 × 10^5.3^ TCID50/mL at 120 hpi, MOI = 0.01) aligned with CPE-based endpoint titration (Figure 1D).

### 3.2. Whole-Genome Sequencing and Phylogenetic Analysis

**Figure 2:**
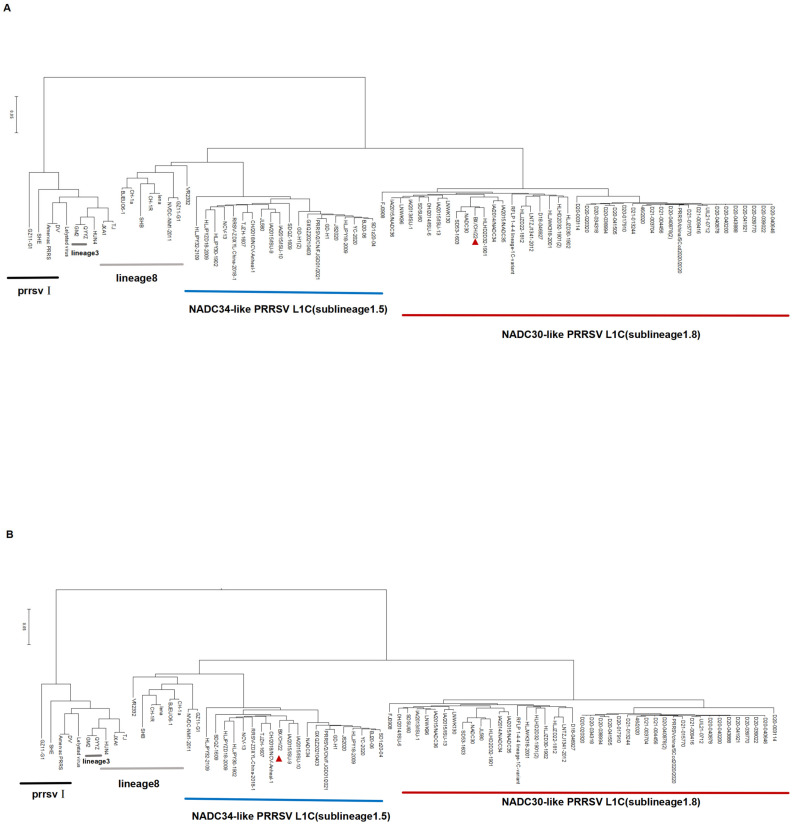
Phylogenetic analysis of the BX/CH/22 strain. (**A**) Phylogenetic tree constructed based on ORF 5. (**B**) Phylogenetic tree constructed based on the full-length genome. The red arrow indicates the target strain.

The whole genome of BX/CH/22 was sequenced at 15,033 bp. To establish the genetic relationship between BX/CH/22 and the other PRRSV isolates, a phylogenetic tree was constructed based on the ORF 5 gene sequence. Analysis of the ORF 5 gene sequence revealed that BX/CH/22 forms a separate clade (Figure 2A) with the NADC30-like PRRSV strain assigned to L1.8. A phylogenetic tree was constructed based on the whole genome sequence of BX/CH/22, which we found to be classified as lineage 1.5 (Figure 2B). The phylogenetic tree was reconstructed using MAFFT-aligned complete genomes with gap-containing sites excluded. The large homology differences between some strains may be related to the presence of additional recombination fragments in some strains. Therefore, the BX/CH/22 strain is a good model for assessing the pathogenicity of multiple recombinant strains.

### 3.3. Recombination Analysis

**Figure 3:**
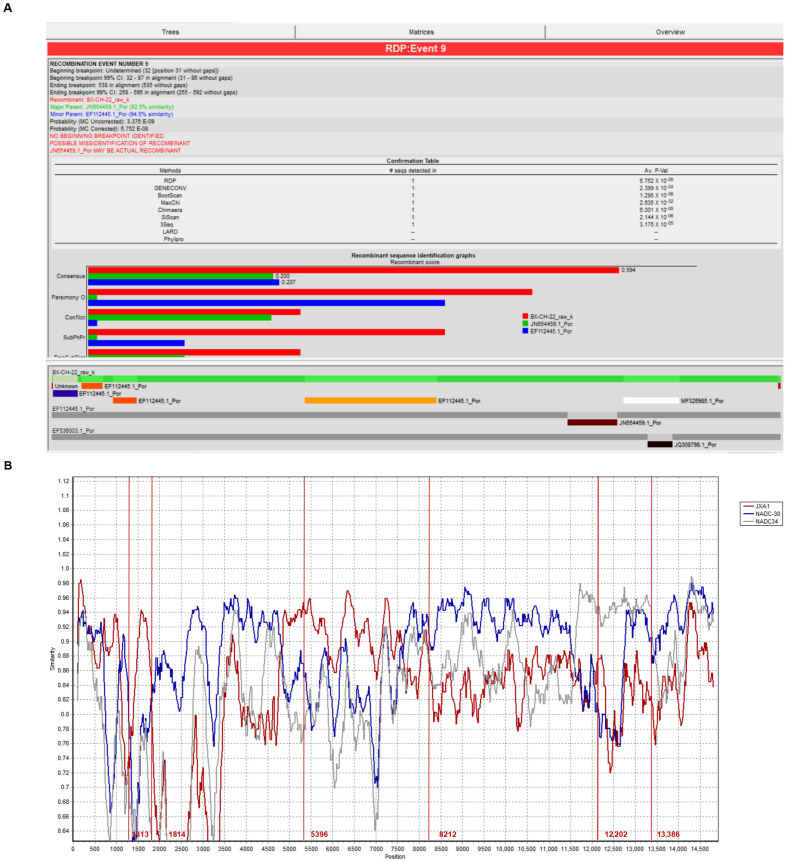

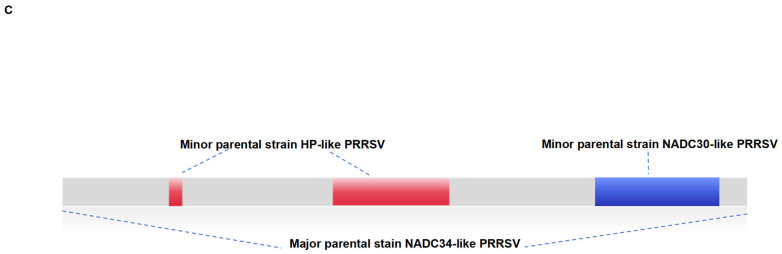
(**A**) Prediction of reorganization events using RDP4 software (version RDP4.46). (**B**) Recombination analysis of the BX/CH/22 strain. The complete genome of BX/CH/22 was selected as the query sequence. The solid red line indicates the reorganization breakpoint; the locus is shown at the bottom. The sequences between the solid lines are the secondary parental region, while the rest is the primary parental region. (**C**) Structure of recombination pattern of BX/CH/22.

Recombination analysis of BX/CH/22 using SimPlot and RDP 4 showed that BX/CH/22 is a recombinant virus derived from the combination of NADC30-like and NADC34-like PRRSV strains. All strains shared two recombinant fragments (Figure 3A,C). To determine the recombination pattern of BX/CH/22, we further analyzed the recombination patterns of all NADC30-like and NADC34-like PRRSV combination strains with complete genome sequences in the GenBank database. The results indicated that BX/CH/22 is a recombinant virus of JXA1, NADC34, and NADC30, and two recombination events were identified. The main parent strain was NADC34, and NADC30 and JXA1 were secondary parent strains. Recombination occurred at 1313–1814 nt, 5396–8212 nt, and 12,202–13,386 nt (Figure 3B).

### 3.4. Clinical Signs

**Figure 4:**
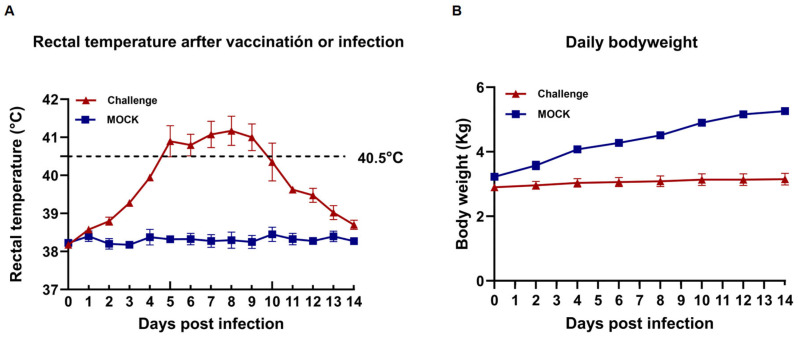
Clinical signs in BX/CH/22-infected piglets. (**A**) Change in body temperature. (**B**) Change in body weight. The dotted line in the figure indicates the upper limit of normal body temperature for piglets.

All piglets infected with BX/CH/22 showed significant clinical signs, including persistent fever, cough, anorexia, and lethargy (Figure 4A,B). All 4 piglets in the challenge group had persistent fever for 3–10 dpi. Further, at 4–9 dpi, some piglets had a body temperature over 40.5 °C; the body temperature of these piglets returned to normal at 11 dpi (Figure 4A). The control piglets did not develop any clinical signs in animals throughout the trial. The body weights of the piglets were recorded on alternate days after the challenge; the results showed that the mean daily weight gain was significantly lower in BX/CH/22-infected piglets between 8 and 14 dpi (*p* < 0.001) (Figure 4B). All the piglets survived until the end of the experiment.

### 3.5. Anti-PRRSV Antibody Detection

**Figure 5:**
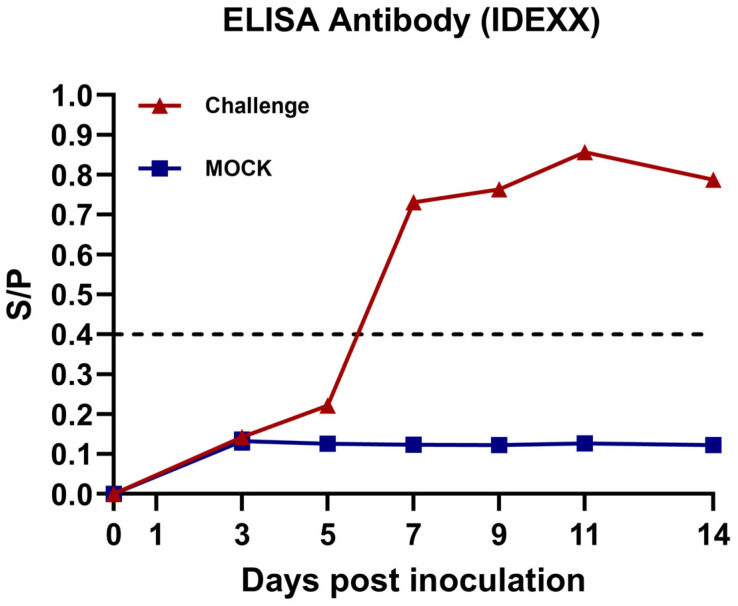
PRRSV N-protein antibody S/P ratio in BX/CH/22-infected piglets. The dotted line in the figure indicates the upper limit of the S/P value, and exceeding it is positive.

Serum samples were collected from the piglets, and the anti-PRRSV N-protein antibody levels were tested. All the piglets in the challenge group were seroconversion positive at 7 dpi. Over time, the anti-PRRSV N-protein antibody levels gradually increased before 11 dpi and decreased at 14 dpi (Figure 5). In contrast, the animals in the control group consistently remained seronegative throughout the experiment.

### 3.6. Gross Pathology and Histopathology

**Figure 6:**
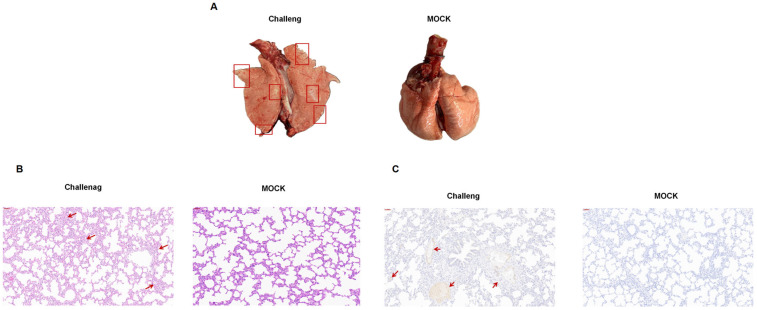
Histopathological lesions in lung tissues of piglets after infection with the BX/CH/22 strain. (**A**) Photographs of lung necropsy. (**B**,**C**) Hematoxylin and eosin staining and immunohistochemistry of lung tissues. The red box area and the arrows indicate the location of the lung tissue lesion.

All experimental animals were euthanized and necropsied at 14 dpi. Compared to the control group, the challenged piglets showed lesions typical of PRRSV infection (Figure 6A). Histopathological examination revealed pulmonary interstitial edema and severe interstitial pneumonia in the animals in the challenge group (Figure 6B). Furthermore, submandibular lymph node enlargement was observed in the challenge group (Figure 6C). No pathological changes to the abovementioned tissues were detected in the control piglets.

### 3.7. Assessment of Viremia and Viral Loads in Tissue and Serum

**Figure 7:**
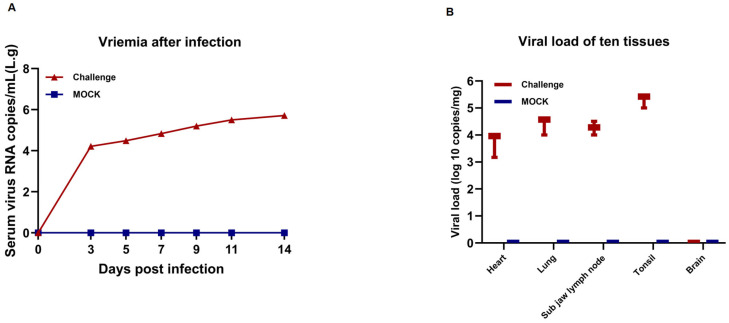
Viral load in BX/CH/22-infected piglets. (**A**) Viral load in selected tissues (heart, lung, lymph nodes, tonsil, and brain). (**B**) Viral load in blood. Data represent mean ± SEM (*n* = 4 per group).

Viral loads in the piglet serum samples at 0, 3, 5, 7, 9, 11, and 14 dpi and animal tissues were analyzed by RT-qPCR. The results showed that the blood viral load of piglets increased rapidly between 0 and 3 dpi after BX/CH/22 infection and slowed down after 4 dpi. However, persistent viremia was observed throughout the experiment (Figure 7A). No viremia was observed in the animals from the control group. BX/CH/22 was detected using viral load measurements in postmortem animal tissues, including the heart, lung, mandibular lymph node, and tonsil. However, BX/CH/22 was not detected in the brain. The highest viral load was detected in the tonsils (Figure 7B).

## 4. Discussion

Since the emergence of the HP-PRRSV strain in China in 2006 [18], it has markedly affected the pig industry. Since 2013, NADC30-like strains have circulated and caused epidemics in many provinces in China. These strains have become the main circulating strains of PRRSV [19]. In 2017, NADC34 PRRSV was discovered for the first time in China [20,21]. By 2021, NADC34 PRRSV, HP-PRRSV, and NADC30 PRRSV were the three most prevalent strains of PRRSV in China [22]. These strains produced many recombinant viruses in a short period, facilitating their rapid spread across China. Viral recombination plays a crucial role in viral epidemics and infectious disease outbreaks, as recombination can increase the genetic diversity of viruses [23], enhance the adaptability of viruses to the environment, improve replication efficiency, and promote the spread of viruses by evading host immune surveillance [24]. In recent years, a large number of clinical samples have been collected to investigate the prevalence of PRRSV [25]. Among the clinical samples collected, we isolated a strain with two recombinant fragments and named it BX/CH/22. Whole-genome sequencing of BX/CH/22 showed that its sequence backbone was dominated by the NADC34-like strain, with recombinant fragments from the JXA1 and NADC30-like PRRSV strains. BX/CH/22 was classified as NADC30-like based on ORF 5 phylogenetic analysis and NADC34-like based on genome-wide sequence analysis. NSP2 is a variable region in the PRRSV genome, and it has been reported that HP-PRRSV NSP2 is missing 30 amino acids, while NADC30 NSP2 is missing 131 amino acids, and NADC34-like NSP2 is missing 100 continuous amino acids. By analyzing the NSP2 amino acid sequence of BX/CH/22, we found that the amino acid deletion pattern in the NSP2 gene enhanced its similarity with JXA1. GP5 gene sequence analysis showed that the isolates formed a separate branch on the NADC30-like PRRSV, and these mutations may alter antigenic properties and host-cell interactions due to GP5-mediated receptor binding and antibody neutralization. The recombination of PRRSV in these regions may be associated with enhanced replication capacity and cellular tropism. These survival and transmission determinants correlate with suboptimal vaccine-induced immunity in swine [11].

Due to the widespread use of the JXA1-R vaccine, it is speculated that the recombinant fragment of JXA1 may have originated from the virus from this vaccine, while fragments from NADC30 and NADC34 were derived from wild strains [26]. According to the accumulated data, the pathogenicity of recombinant PRRSV strains is generally intermediate between parent strains [10]. Most recombinant NADC34 strains exhibit moderate pathogenicity [27]; however, highly pathogenic recombinant strains may also occur, as evidenced by the existence of the recombinant HP-PRRSV strain [28]. We further investigated whether the recombination of this gene fragment affected the pathogenicity of the strain. Animal challenge experiments showed that piglets inoculated with BX/CH/22 showed moderate typical clinical signs in animals. Viral load measurements in autopsy tissues showed the presence of BX/CH/22 in all tissues except the brain. In addition, the highest viral load was detected in the tonsils of piglets in the challenge group. It is hypothesized that this strain does not have the ability to cross the blood–brain barrier and accumulates in the tonsils, similar to the NADC30 strain. Therefore, it can be speculated that BX/CH/22 can cause long-term viremia and severe histopathological changes. However, the test piglets survived throughout the trial cycle. Persistent viremia was exhibited throughout the experiment. The results of H&E staining and immunohistochemistry showed that the infected piglets showed evident pathological changes in the lungs, suggesting that the isolate can have a highly damaging effect on lung tissues. Therefore, BX/CH/22 affects piglet growth and performance but is significantly less pathogenic than HP-PRRSV. We speculate that the reduced pathogenicity is linked to the genetic recombination ability of the strain. The unique recombination pattern involving a vaccine strain suggests a potential mechanism for the isolate’s divergent traits; however, further functional studies are required to establish a causal relationship between these recombination events and pathogenicity. In future studies, we aim to determine how recombination of this gene fragment affects the pathogenicity of the strain.

In conclusion, we isolated and reported a new NADC34-like PRRSV. While its associated mortality and clinical characteristics are similar to those of circulating strains in the wild, large-scale transmission is still possible due to factors such as environmental influences and secondary infections. Therefore, the emergence of a unique recombination pattern of three recombination events raises concerns about the epidemiology of PRRSV, which may pose new threats and challenges to the pig industry, requiring continuous monitoring of potential risks.

## Data Availability

The data that support the findings of this study are available from the corresponding author upon reasonable request.

## Abbreviations

The following abbreviations are used in this manuscript:

BSA	bovine serum albumin
CPE	cytopathic effect
dpi	days post-infection
H & E	hematoxylin and eosin
hpi	hours post-infection
NSP	non-structural protein
ORF	open reading frame
PRRSV	porcine reproductive and respiratory syndrome virus
PRV	pseudorabies virus
CSFV	classical swine fever virus
RT-PCR	real-time polymerase chain reaction
TCID50	50% tissue culture infectious dose
